# Japanese encephalitis virus infection induces mitochondrial-mediated apoptosis through the proapoptotic protein BAX

**DOI:** 10.3389/fmicb.2024.1485667

**Published:** 2024-10-28

**Authors:** Ke Yang, Xinran Li, Shuqing Yang, Yi Zheng, Sanjie Cao, Qigui Yan, Xiaobo Huang, Yiping Wen, Qin Zhao, Senyan Du, Yifei Lang, Shan Zhao, Rui Wu

**Affiliations:** ^1^Research Center of Swine Disease, College of Veterinary Medicine, Sichuan Agricultural University, Chengdu, China; ^2^Sichuan Science-Observation Experiment Station of Veterinary Drugs and Veterinary Diagnostic Technology, Ministry of Agriculture, Chengdu, China

**Keywords:** JEV, mitochondrial apoptotic pathway, BAX, P53-BAX pathway, PK-15

## Abstract

The Japanese encephalitis virus (JEV), a zoonotic flavivirus, is Asia’s primary cause of viral encephalitis. JEV induces apoptosis in a variety of cells; however, the precise mechanisms underlying this apoptosis resulting from JEV infection remain to be elucidated. Our previous studies showed that the proapoptosis gene BAX may have a role in JEV proliferation. In this study, we constructed a PK-15 cell line (BAX.KO) with a knockout of the BAX gene using CRISPR/Cas9. The knockout of the BAX gene effectively inhibited the proliferation of JEV, resulting in a 39.9% decrease in viral protein levels, while BAX overexpression produced the opposite effect. We confirmed that JEV induces apoptosis of PK-15 using 4′,6-diamidino-2-phenylindole (DAPI) staining and Annexin V-FITC/PI staining. Furthermore, we found that the phosphorylation of P53 and the expression levels of BAX, NOXA, PUMA, and cleaved-caspase-3/9 were significantly upregulated after JEV infection. Moreover, we found that JEV infection not only caused mitochondrial damage, the release of mitochondrial cytochrome C (Cyt C), and the downregulation of the apoptosis-inhibiting protein BCL-2 but also reduced the mitochondrial membrane potential (MOMP) and the accumulation of intracellular reactive oxygen species (ROS). These factors collectively encourage the activation of the mitochondrial apoptosis pathway. In contrast, BAX gene knockout significantly reduces the apoptotic changes caused by JEV infection. Treatment with the caspase3 inhibitor attenuated JEV-induced viral proliferation and release, leading to a decrease in viral protein levels of 46% in PK-15 cells and 30% in BAX.KO cells. In conclusion, this study clarified the molecular mechanisms of JEV-induced apoptosis and provided a theoretical basis for revealing the pathogenic mechanisms of JEV infection.

## 1 Introduction

JEV belongs to the genus *Flavivirus* of the family *Flaviviridae* and is a mosquito-borne virus (mainly transmitted by *Culex pipiens*). It is a positive-sense, single-stranded RNA virus (Sharma et al., 2021; Quan et al., 2023). Japanese encephalitis (JE) is a pathogenic viral encephalitis caused by the JEV (Lopez et al., 2015). According to the 2019 WHO Japanese Encephalitis Fact Sheet, there are an estimated 68,000 clinical cases and 1,000–2,000 deaths from JE each year. The incubation period is 5–15 days, sometimes, it is up to several weeks, and approximately 50–80% of those infected are asymptomatic. Common symptoms include fever, headache, nausea, and vomiting, as well as neurological symptoms such as paralysis and movement disorders. In severe cases, epileptic seizures may occur, and some patients may still experience long-term sequelae during the recovery process after the acute phase (Solomon et al., 2000; Solomon, 2004; Misra and Kalita, 2010). Pigs, as carriers of the JE virus, can transmit the virus through mucus contact and corresponding droplet-unmediated transmission, allowing the virus to continue to spread during the winter months when there are fewer mosquitoes (So Lee et al., 2018). As the number of farmed animals increases globally, the expansion of pig farming may raise the risk of JEV transmission and increase the likelihood of epidemic outbreaks among humans (James et al., 2018). Therefore, it is necessary to explore the pathogenic mechanisms of JEV to design better disease control strategies.

Apoptosis, also known as programmed cell death (PCD), is a self-protection mechanism used by multicellular organisms to clear senescent, damaged, or pathogen-infected cells (Damien et al., 2021). Apoptotic cells are characterized by membrane blebbing, decreased cell size, nuclear fragmentation, chromatin condensation, exposure of phosphatidylserine (PtdSer) on the cell surface, and apoptotic body formation (Peter, 2015). Apoptosis is induced by two classical pathways: the intrinsic and extrinsic pathways (Avi and Guy, 2014). The intrinsic pathway is also known as the mitochondrial apoptotic pathway, in which mitochondria act as the major players. Various triggers can trigger mitochondrial apoptosis, such as DNA damage, growth factor, or nutrient deprivation. These stimuli activate the proapoptotic protein BAX, which oligomerizes at the mitochondria and undergoes a series of conformational changes that lead to its incorporation into the mitochondrial membrane, ultimately inducing apoptosis (Kurt et al., 2002; Liying and Donald, 2008; Czabotar et al., 2013). Death receptors on the plasma membrane activate the extrinsic pathway and lead to the activation of caspase-8 (Carneiro and El-Deiry, 2020). Upon activation of initiators caspase-8 and -9, the two apoptotic pathways converge at the proteolytic activation step of effectors caspases-3 and -7, thereby inducing apoptosis (Caroppi et al., 2009; Ketelut-Carneiro and Fitzgerald, 2022).

Members of the B cell lymphoma 2 (BCL-2) protein family are critical regulators with pro- and antiapoptotic activities (Shanna et al., 2022). The BCL-2 family is divided into three distinct groups based on the presence of the BCL-2 homology (BH) domain. These include full-length anti-apoptotic proteins (BCL-2 and BCL-XL), full-length proapoptotic proteins (BAX and BAK) that contain all four BH domains, and proapoptotic activating proteins that contain only the BH3 domain (BH3-only proteins, such as BIM, BBC3, BID, BAD, and NOXA) (Mark and Jerry, 2015; Pan et al., 2021). Studies have shown that BAX can regulate the timing and extent of cell death in cells infected with various viruses, release viral particles, and achieve viral proliferation (Suzuki et al., 2018).

Viruses can regulate their proliferation by promoting or inhibiting apoptosis of host cells. Over the years, viruses have evolved strategies that modulate host apoptotic responses to facilitate their survival and propagation (Lorenzo et al., 2008). Previous studies have reported that BCL-2 family proteins control mitochondrial-mediated apoptosis in flaviviruses (dengue virus, Zika virus, and West Nile virus) to maintain cellular homeostasis by eliminating damaged cells and pathogen-infected cells (Bimmi et al., 2003; Junqi et al., 2014; Lukasz et al., 2017). However, as a member of the Flavivirus family, whether JEV infection induces apoptosis through the BAX-mediated mitochondrial apoptosis pathway is a question of concern. In this study, we found that BAX plays an essential role in JEV infection. JEV achieves viral proliferation through the mitochondrial apoptosis pathway. Blocking mitochondrial apoptosis can effectively inhibit the proliferation of JEV.

## 2 Materials and methods

### 2.1 Cells, viruses, and antibodies

Porcine kidney cells (PK-15), PK-15 cells with BAX gene knockout (BAX.KO), human embryonic kidney cells (HEK-293T), and baby hamster kidney cells (BHK-21) were cultured in Dulbecco’s modified Eagle’s medium (DMEM; Gibco, USA) supplemented with 10% fetal serum (FBS; HyClone, South Logan, USA) and 1% penicillin–streptomycin at 37°C in a 5% CO_2_ incubator.

The SCYA201201-1 strain of JEV (GenBank accession number: KU508408.1) was provided by the Swine Disease Research Center, College of Veterinary Medicine, Sichuan Agricultural University.

Several primary antibodies were used for Western blotting at a dilution of 1:2,000: Anti-E (HL2517) from Gene Tex (San Antonio, USA); Anti-β-actin (AC026), HRP-conjugated goat anti-rabbit (AS014), and HRP-conjugated goat anti-mouse (AS003) from Abclonal (Wuhan, China); Anti-BAX (50599-2-Ig), anti-EGFP (50430-2-AP), and anti-P53 (60283-2-Ig) from Proteintech (Wuhan, China); Anti-pP53 (9284) from Cell Signaling Technology, Inc. (USA); Anti-caspase-9 (ab185719) and anti-caspase-3 (ab32150) from Abcam (Cambridge, MA, USA); Anti-γ-H2AX (C2035S) from Beyotime Biotechnology (Shanghai, China); and Anti-Cytochrome C (SC69-08) and anti-BCL-2 (HA721235) from Huabio (China).

### 2.2 Plasmids and primers

psPAX (12260), pMD2.G (12259), and LentiCRISPR-V2 (52961) were purchased from Addgene. The full-length BAX gene was cloned into the pEGFP vector with an EGFP tag at the C-terminus to generate the expression plasmid pEGFP-BAX (GenBank accession: XM_003127290.5). The primers used for plasmid construction and real-time quantitative reverse transcription polymerase chain reaction (RT-qPCR) in this study are listed in Table 1. The sequences of the recombinant plasmids were verified using Sanger dideoxy sequencing.

**Table 1:** Primer pairs were used in this study.

Gene	Sequence (5′ − 3′)	Application
pEGFP-BAX-F	agtactcagatctcgagATGGACGGGTCCGGGGAG	Cloning
pEGFP-BAX-R	cgtcgactgcagaattcTCAGCCCATCTTCTTCCAGA	Cloning
PUMA-F	AGGCCCTCTACGGGCTC	RT-qPCR
PUMA-R	CTTCTGGAGCGTGTCCCATC	RT-qPCR
NOXA-F	TCAGGTTCCTAGGCGGAAGA	RT-qPCR
NOXA-R	CCTGAAGTCGAGTGTGCCAT	RT-qPCR
JEV-E-F	CAGTGGAGCCACTTGGGTG	RT-qPCR
JEV-E-R	TTGTGAGCTTCTCCTGTCG	RT-qPCR
β-actin-F	CCTTGATGTCCCGCACG	RT-qPCR
β-actin-R	GCTGTCCCTGTACGCCTCTG	RT-qPCR
ND1-F	TAGGATGATTGCCAGTGTTACTTCA	RT-qPCR
ND1-R	TCCACTACCAATACCCTACCCTCTA	RT-qPCR
RPLP0-F	GGCAGCATCTACAACCCTGAAGTG	RT-qPCR
RPLP0-R	CCCATATCCTCGTCCGACTCCTC	RT-qPCR

qRT-PCR, real-time quantitative reverse transcription polymerase chain reaction.

### 2.3 Western blot

Cells were washed twice with cold PBS and then incubated for 20 min at 4°C in a RIPA lysis buffer with phenylmethylsulfonyl fluoride (PMSF) (Solarbio, Beijing, China). Lysates were centrifugated at 12,000 rpm for 5 min at 4°C, and the supernatants were collected. The supernatants were mixed with a protein loading buffer and boiled at 100°C for 10 min. After cooling, the bicinchoninic acid (BCA) concentration of the protein was determined, and the total amount of protein loaded was unified. Proteins were separated using 10% sodium dodecyl sulfate–polyacrylamide gel electrophoresis (SDS-PAGE) and then transferred to polyvinylidene fluoride membranes (Bio-Rad, Hercules, CA, USA). After blocking in tris-buffered saline with 0.1% Tween^®^ 20 detergent (TBST) containing 5% skimmed milk powder (A600669, Sangon) at room temperature for 2 h, the membranes were incubated with different primary antibodies overnight at 4°C. All antibodies were diluted in a primary antibody dilution buffer (Beyotime, Shanghai, China). The membranes were washed four times with TBST for 4 min each and incubated with 1:5000 HRP-conjugated goat anti-rabbit immunoglobulin G (IgG) for 45 min at room temperature. The membranes were rewashed, and bands were developed by adding ECL (Bio-Rad, Hercules, CA, USA) according to the manufacturer’s instructions.

### 2.4 Detection of mtDNA copies and mitochondrial membrane potential

BAX.KO and PK-15 cells were infected with JEV (multiplicity of infection [MOI] = 1) for 12 or 24 h, and cellular DNA was extracted and used as a template after a 50-fold dilution. Cytoplasmic mtDNA levels were determined using RT-qPCR with the TransStart® Top Green qPCR SuperMix (+Dye I) Kit (AQ132-11, TransGen Biotech).

BAX.KO and PK-15 cells were infected with JEV (MOI = 1) for 24 h and then stained with 2 μg/mL JC-1 dye (C2006, Beyotime Biotechnology) for 30 min. After three washes with dye buffer, the fluorescence intensity of JC-1 monomers (green fluorescence) vs. JC-1 aggregates (red fluorescence) was observed using a fluorescence microscope (Olympus BX63, Tokyo, Japan).

### 2.5 Reactive oxygen species assay

BAX.KO and PK-15 cells were infected with JEV (MOI = 1) for 36 h. The DCFH-DA probe (Beyotime Biotechnology, Shanghai, China) was diluted to 10 μmol/L in serum-free DMEM and added to the cells for a 20-min incubation. After the samples were washed with serum-free DMEM, the fluorescence signal was immediately detected at an excitation wavelength of 488 nm and an emission wavelength of 525 nm.

### 2.6 Construction of the BAX gene knockout PK-15 cell line using the CRISPR/Cas9 system

Specific sgRNAs targeting the BAX gene (sgRNA-F: 5′-CACCGAGCGAGTGTCTCAAGCGCAT-3′ and sgRNA-R: 5′-AACATGCGCTTGAGACACTCGCTC-3′) were designed using an online CRISPR tool.^1^ Two sgRNA oligonucleotides were inserted into the LentiCRISPR-V2 plasmid. The recombinant plasmids were co-transfected into HEK-293T cells with psPAX and pMD2.G using Lipofectamine 3000 (Invitrogen, Carlsbad, CA, USA). The supernatant was filtered and collected as a lentiviral solution containing the target sgRNA to select positive cells 24 h post-infection. The BAX knockout cell line was confirmed using DNA sequencing and Western blotting, and after checking the difference with PK-15 cells, the monoclonal cell line was selected for subsequent experiments.

### 2.7 EdU cell proliferation assay

BAX.KO and PK-15 cells were seeded into 12-well plates. Furthermore, 5-ethynyl-2′-deoxyuridine (EdU) cell proliferation assays were conducted using the BeyoClick™ EdU Cell Proliferation Kit with Alexa Fluor 555 (Beyotime, Shanghai, China) in accordance with the manufacturer’s instructions. The nuclei were stained with DAPI (Beyotime, Shanghai, China) at room temperature for 10 min. The stained cells were then observed under a fluorescence microscope. The proportion of EdU-positive cells was calculated using ImageJ software.

### 2.8 RNA extraction and RT-qPCR

Total RNA was extracted using the Trizol Total RNA Isolation Kit (Sangon Biotech, Shanghai, China) and then reverse transcribed with the PrimeScript™ RT Reagent Kit (Takara Bio, Tokyo, Japan). Synthetic cDNA was analyzed for quantitative real-time PCR using TB Green Premix Ex Taq™ II (Tli RNaseH Plus) (TaKaRa Bio) in a LightCycler 96 System (Roche, Basel, Switzerland). PCR amplification was carried out by denaturation at 94°C for 30 s followed by 40 cycles of 94°C for 15 s and 60°C for 30 s while the melting curve analysis was performed at 60–95°C. The ΔΔCt method was used to measure the expression levels of target genes. Data were normalized to the level of the control gene encoding β-actin and showed a fold change by normalizing to the mock control.

### 2.9 Statistical analysis

Three times each of the tests were repeated. The information was statistically analyzed using the GraphPad Prism 8 program and is shown as the mean ± standard deviation (SD). Unpaired *t*-tests were used for comparisons between two sets of data, and one-way analyses of variance (ANOVA) were used for comparisons between several groups. The differences were deemed significant at *p* < 0.05.

## 3 Results

### 3.1 Construction of BAX gene knockout PK-15 cell line

**Figure 1:**
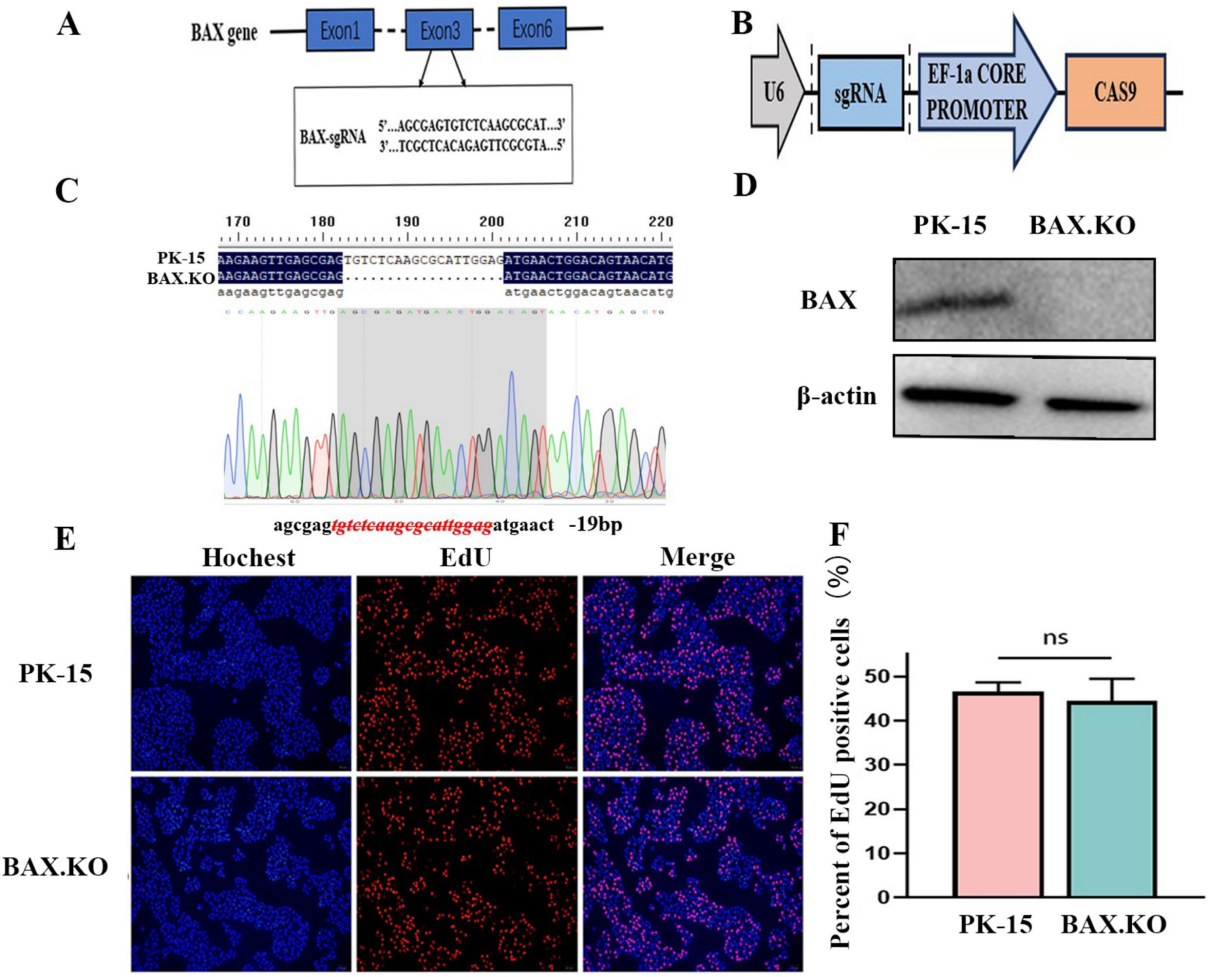
CRISPR/Cas9 gene editing technology knocked out the BAX gene in PK-15 cells. **(A)** The sgRNA sequences targeted exon 3 of the BAX gene. **(B)** Schematic illustration of the LentiCRISPR V2 plasmid with a restriction site (BsmBI) existing in the target gene. **(C)** DNA sequencing results of PK-15 and BAX.KO cells. **(D)** Detection of BAX protein knockout efficiency by Western blot analysis. **(E)** PK-15 and BAX.KO cells incubate for 2 h after EdU loading. After the click reaction, pictures were taken using a fluorescence microscope. 5-Ethynyl-2′-deoxyuridine (EdU)-positive cells were shown in red, and the nuclei were shown in blue. **(F)** At least three photos were taken of each sample to calculate the percentage of EdU-positive cells, with the average reported as the final data. ns, not significant.

The designed sgRNA is shown in Figure 1A, which originates from the third exon of the BAX gene sequence. The double-stranded sgRNA was ligated to the linear LentiCRISPR V2 plasmid digested by the BsmB I enzyme (Figure 1B). The PK-15 cell line with BAX gene knockouts constructed using CRISPR/Cas9 was named BAX.KO cells. Genomic DNA was extracted from the PK-15 and BAX.KO cells and subjected to sequence analysis. Figure 1C illustrates that a 19-bp deletion occurred at the BAX gene target site, indicating that the Cas9 protein successfully sheared the target DNA, resulting in random frameshift mutations in the sequence. Western blot analysis confirmed that the BAX protein was knocked out (Figure 1D). These results confirm that the BAX gene was successfully knocked out, and this knockout did not affect cell proliferation (Figures 1E,F).

### 3.2 Knockout of BAX inhibits the proliferation of JEV

**Figure 2:**
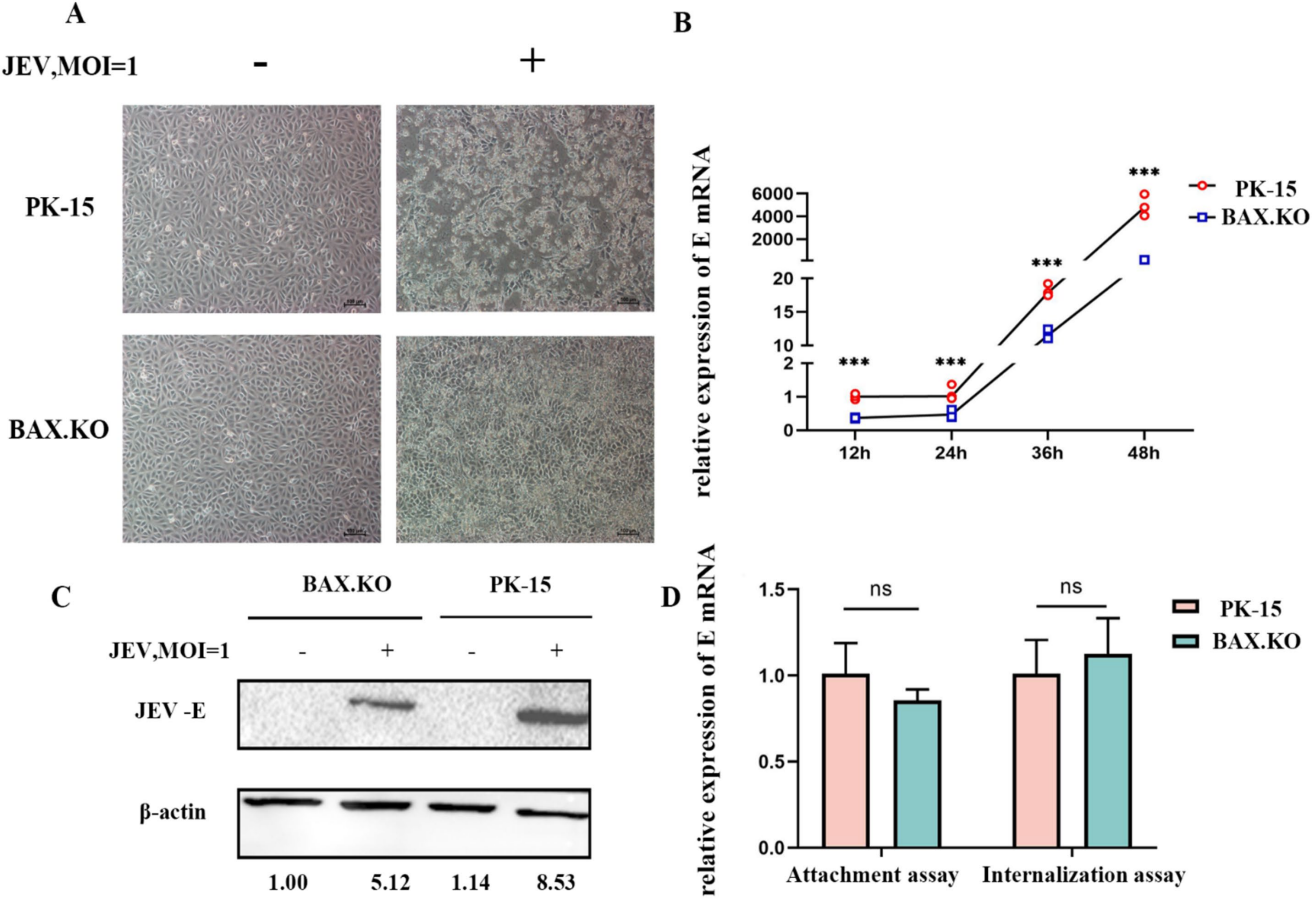
Knockout of BAX gene inhibits the proliferation of Japanese encephalitis virus (JEV) in PK-15 cells. **(A)** Observation of cell status changes 36 h after JEV infection (multiplicity of infection [MOI] = 1). **(B)** Infected PK-15 and BAX.KO cell samples were collected at the indicated time, and relative fluorescence quantitative RT-qPCR was performed to detect changes in E genes, and growth curves were drawn. **(C)** PK-15 and BAX.KO cells were infected with JEV (MOI = 1) for 36 h; the Western blot technique was performed to analyze E protein and quantitative analysis was performed using ImageJ. **(D)** Role of BAX in JEV binding and cellular internalization. Virus adsorption to cells and cellular internalization were measured using real-time quantitative reverse transcription polymerase chain reaction (RT-qPCR) of the JEV-E gene. ***p < 0.001. ns, not significant.

To accurately determine the proliferation kinetics of JEV in BAX.KO and PK-15 cells, the BAX.KO and PK-15 cells infected with JEV (MOI = 1) were collected to monitor cell morphological changes at the specified time. As shown in Figure 2A, the damage to PK-15 cells was observed under the microscope, characterized by cell shrinkage, unclear edges, and cell lysis. In contrast, damage to BAX.KO cells was significantly inhibited during the same observation period under microscopy. The E gene was detected using RT-qPCR and Western blot, revealing that the amount of virus in BAX.KO cells was significantly lower than in PK-15 cells (Figures 2B,C). The levels of the JEV-E gene transcript in PK-15 and BAX.KO cells were determined using RT-qPCR. It was found that JEV attachment and internalization into host cells are independent of the function of BAX proteins (Figure 2D).

### 3.3 Increasing BAX expression promotes JEV infection

**Figure 3:**
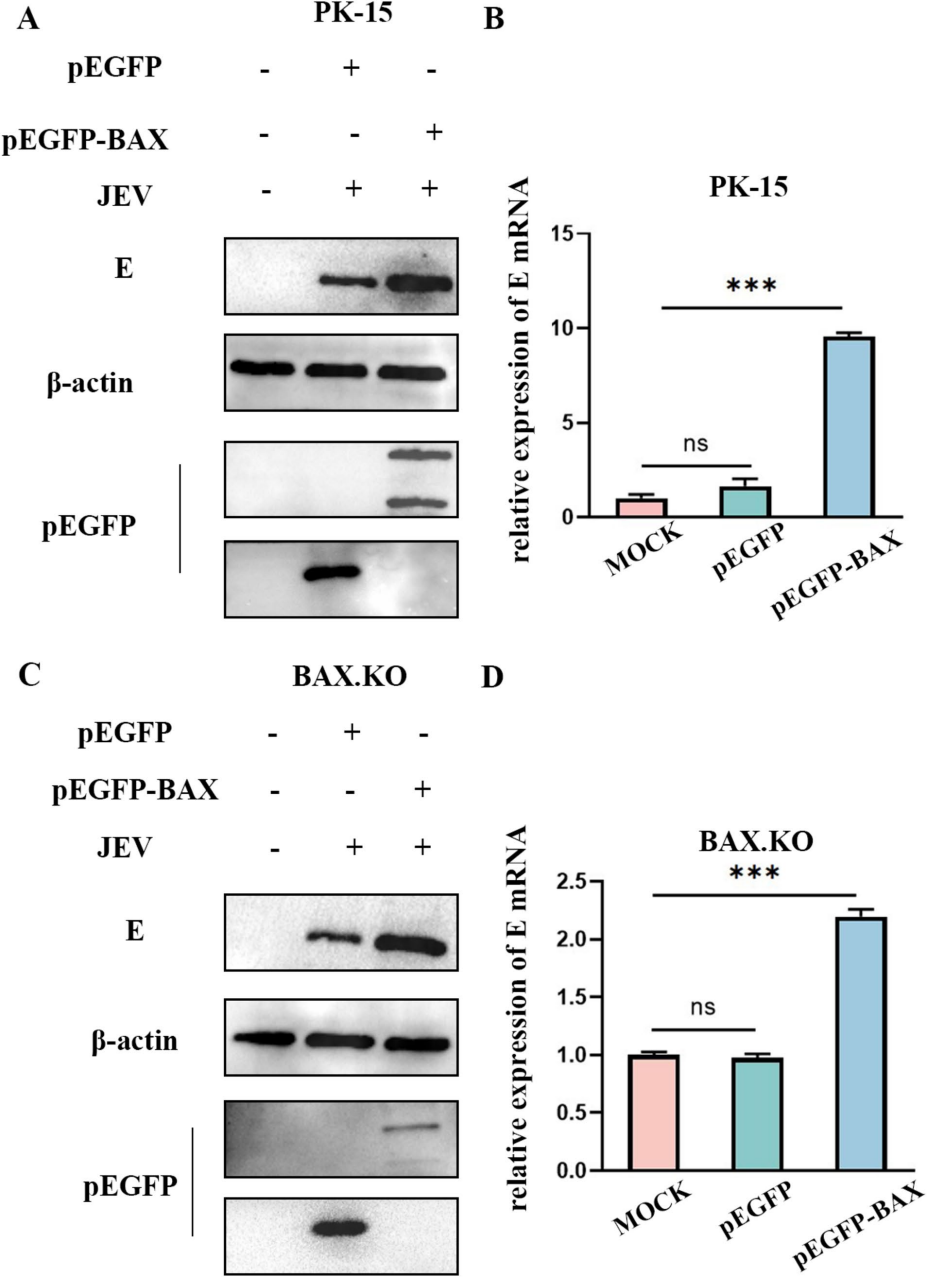
BAX protein plays a positive regulatory role in Japanese encephalitis virus (JEV) proliferation. pEGFP and pEGFP-BAX plasmid were transfected into PK-15 or BAX.KO cells. **(A)** PK-15 cells were infected with JEV (MOI = 1) for 36 h, and the amount of virus was analyzed using Western blot. **(B)** RT-qPCR was performed to measure the level of E mRNA. **(C)** BAX.KO cells were infected with JEV (multiplicity of infection [MOI] = 1) for 36 h, and the amount of virus was analyzed using Western blot. **(D)** Real-time quantitative reverse transcription polymerase chain reaction (RT-qPCR) was performed to measure the level of E mRNA. ***p < 0.001. ns, not significant.

BAX.KO and PK-15 cells were transfected with pEGFP or pEGFP-BAX plasmids, and JEV was inoculated for 36 h (MOI = 1). Figures 3A,C showed that BAX protein was expressed in BAX.KO and PK-15 cells, when the pEGFP-BAX expression vector expresses BAX protein in cells, part of the P21 BAX protein is cleaved by calpain into P18 BAX (Wood and Newcomb, 2000; Xuefang et al., 2003). Compared to control cells, E gene mRNA levels were significantly increased in PK-15 cells overexpressing the BAX gene (Figure 3B). Similarly, BAX gene complementation in BAX.KO cells also increased the mRNA content of the E gene (Figure 3D). Compared to cells transfected with pEGFP plasmid, the JEV-E protein content was higher in cells transfected with pEGFP-BAX plasmid, suggesting that BAX plays a positive regulatory role in JEV proliferation.

### 3.4 Knockout of BAX inhibits apoptosis induced by JEV infection

**Figure 4:**
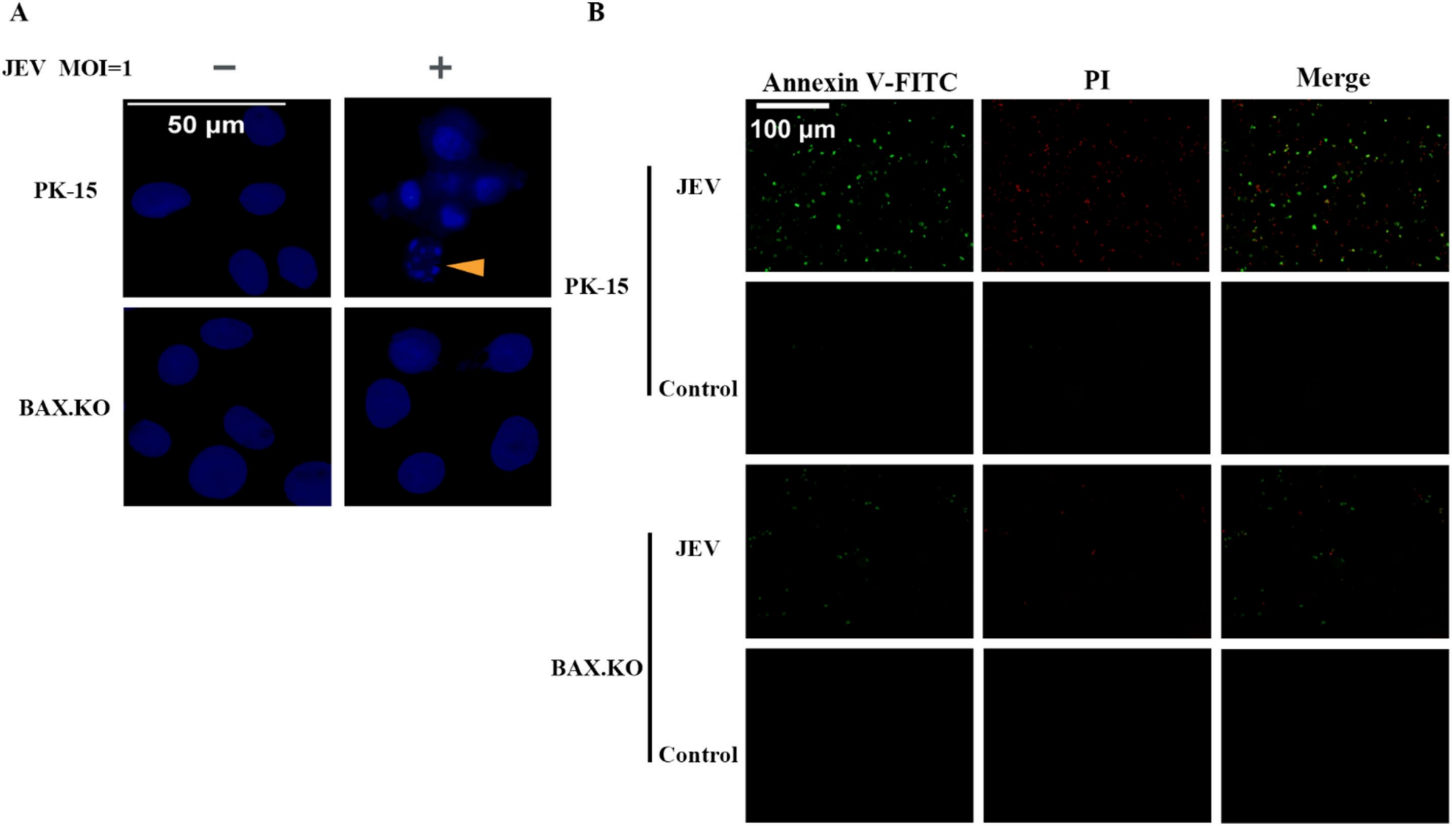
**(A)** Nuclear morphological changes in cells infected with Japanese encephalitis virus (JEV), the arrows indicate that the nuclei of infected cells appeared as typical fragmented and marginated apoptotic bodies. **(B)** Apoptotic cells were observed under a fluorescence microscope.

DAPI staining was performed to observe the morphological changes in the cell nucleus, and nuclear fragmentation formation was observed in the JEV-infected cells, which are denoted by arrowheads (Figure 4A). Nuclear fragmentation and marginalized apoptotic bodies are hallmark changes of apoptosis. In the early stages of apoptosis, cells externalize phosphatidylserine to the cell surface. Annexin V can selectively bind to phosphatidylserine. Using Annexin V labeled with the green fluorescent probe FITC (Annexin V-FITC), we can detect the externalization of phosphatidylserine, and the intensity of green fluorescence can represent the degree of cell apoptosis. JEV infected cells for 24 h (MOI = 1). The cells were treated according to the fluorescence quantitative microscopy observation method of the Annexin V-FITC PI apoptosis and necrosis detection kit and observed under a fluorescence microscope. Cells stained only with green fluorescence were apoptotic cells, and cells stained with green and red fluorescence were necrotic cells (Figure 4B). In summary, JEV infection induced apoptosis of PK-15 cells, and knockout of the BAX gene inhibited JEV-induced apoptosis and viral proliferation.

### 3.5 JEV infection activates mitochondrial apoptosis

**Figure 5:**
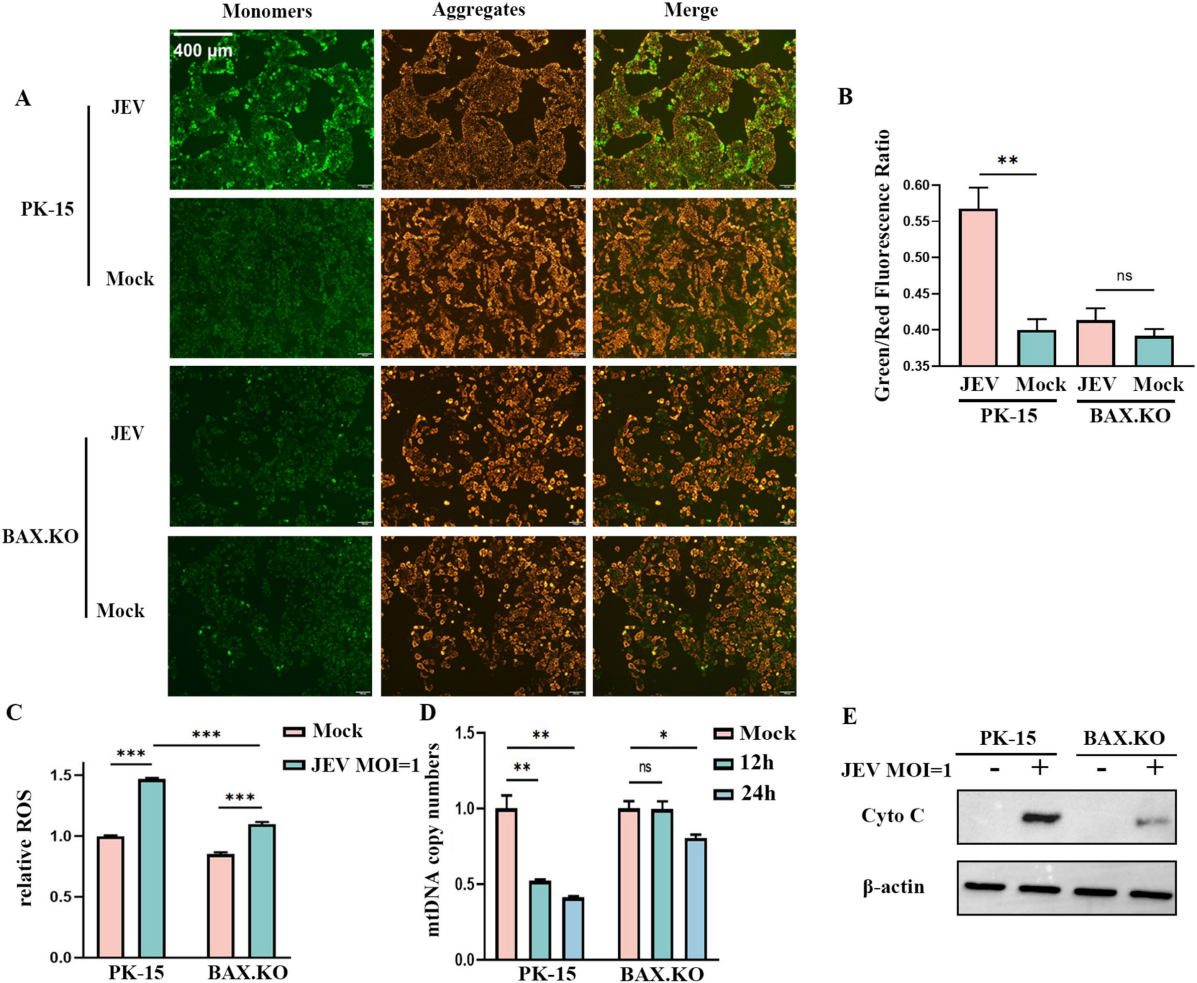
Japanese encephalitis virus (JEV) infection of PK-15 and BAX.KO cells activated the mitochondrial apoptosis pathway, while knockout of BAX alleviated JEV-induced mitochondrial damage. **(A)** Mitochondrial membrane potential (MOMP) was detected using the JC-1 detection kit 24 h after PK-15 and BAX.KO cells were infected with JEV (multiplicity of infection [MOI] = 1). **(B)** The ratio of green fluorescence to red fluorescence indicated the degree of reduction in MOMP, and the fluorescence intensity was quantitatively analyzed using ImageJ. **(C)** PK-15 and BAX.KO cells were loaded with the fluorescent probe DCFH-DA with JEV infection (MOI = 1) for 24 h, and the ROS was measured using a multifunctional microplate reader. **(D)** PK-15 and BAX.KO cells were infected with JEV (MOI = 1) for 12 or 24 h, and the cytoplasmic mtDNA content was determined using real-time quantitative reverse transcription polymerase chain reaction (RT-qPCR). **(E)** PK-15 and BAX.KO cells were infected with JEV (MOI = 1) for 24 h, and the Cyto C protein level was detected using Western blotting. *p < 0.05, **p < 0.01, ***p < 0.001. ns, not significant.

JEV inoculated BAX.KO and PK-15 cells for 24 h (MOI = 1). JC-1 staining working solution was added and incubated at 37°C for 20 min. The changes in mitochondrial membrane potential (MOMP) were detected under a fluorescence microscope. Figure 5A shows that the MOMP of PK-15 was significantly lower than that of BAX.KO. The MOMP was quantitatively analyzed using ImageJ (Figure 5B). The changes in ROS were detected at the peak of JEV proliferation (Figure 5C). BAX.KO and PK-15 cells were inoculated with JEV (MOI = 1) for 36 h, and mtDNA content was detected using qPCR (Figure 5D). Figure 5E shows that JEV infection leads to the release of cytochrome C, a key component of mitochondrial apoptosis. BAX gene knockout can reduce mitochondrial damage caused by JEV infection. In short, by detecting changes in indicators related to mitochondrial apoptosis, it is proven that JEV infection activates mitochondrial apoptosis. Knocking out the BAX gene inhibits a series of changes in mitochondrial apoptosis.

### 3.6 JEV activation of the P53-BAX pathway promotes cell apoptosis

**Figure 6:**
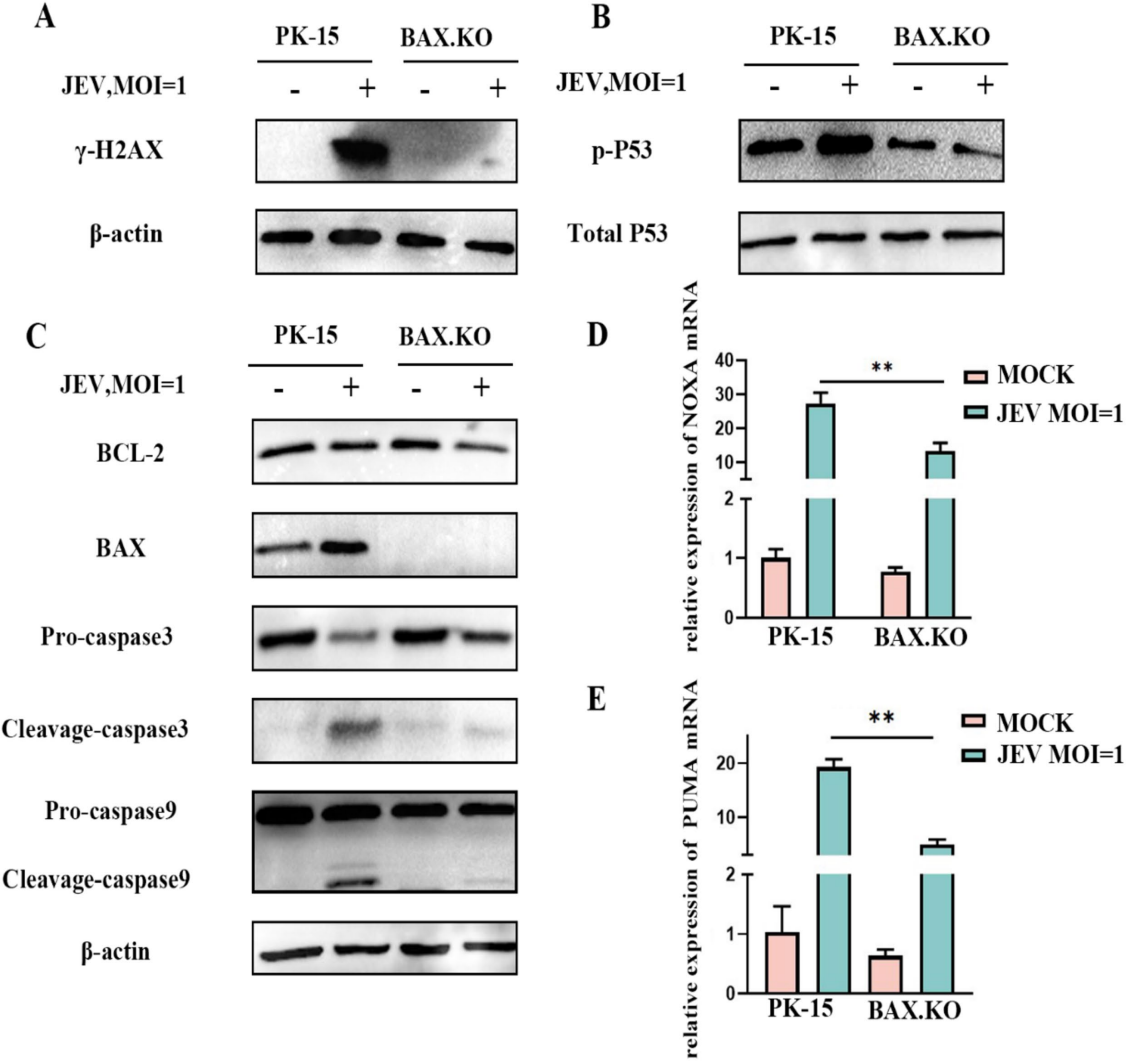
Japanese encephalitis virus (JEV) infection activates proteins related to the mitochondrial apoptosis pathway. PK-15 and BAX.KO cells were infected with JEV (multiplicity of infection [MOI] = 1), and apoptotic proteins related to the mitochondrial apoptosis pathway were verified using Western blotting or real-time quantitative reverse transcription polymerase chain reaction (RT-qPCR). **(A)** γ-H2AX, **(B)** P53 phosphorylation, **(C)** BCL-2, BAX, caspase-3, and caspase-9, **(D)** PUMA mRNA, **(E)** NOXA mRNA, while β-Actin served as loading control. JEV infection of PK-15 cells activated the P53-BAX apoptosis pathway while in BAX.KO cells, the activation of P53-BAX apoptosis pathway proteins was inhibited. **p < 0.01.

BAX.KO and PK-15 cells were inoculated with JEV (MOI = 1), and the expression levels of proteins related to the P53-BAX pathway were detected using Western blotting. JEV infection induces DNA damage (Figure 6A), which in turn promotes P53 phosphorylation (Figure 6B), upregulation of BAX protein, downregulation of BCL-2, cleavage of caspase-9 and caspase-3 (Figure 6C), and upregulation of NOXA and PUMA mRNA (Figures 6D,E). Infection of PK-15 with JEV activates the P53-BAX pathway of the mitochondrial apoptosis pathway to induce cell apoptosis, while knockout of the BAX gene can effectively reduce the changes in a series of apoptotic proteins caused by JEV infection and inhibit cell apoptosis.

### 3.7 Caspase-3 inhibitor inhibits virus proliferation and release

**Figure 7:**
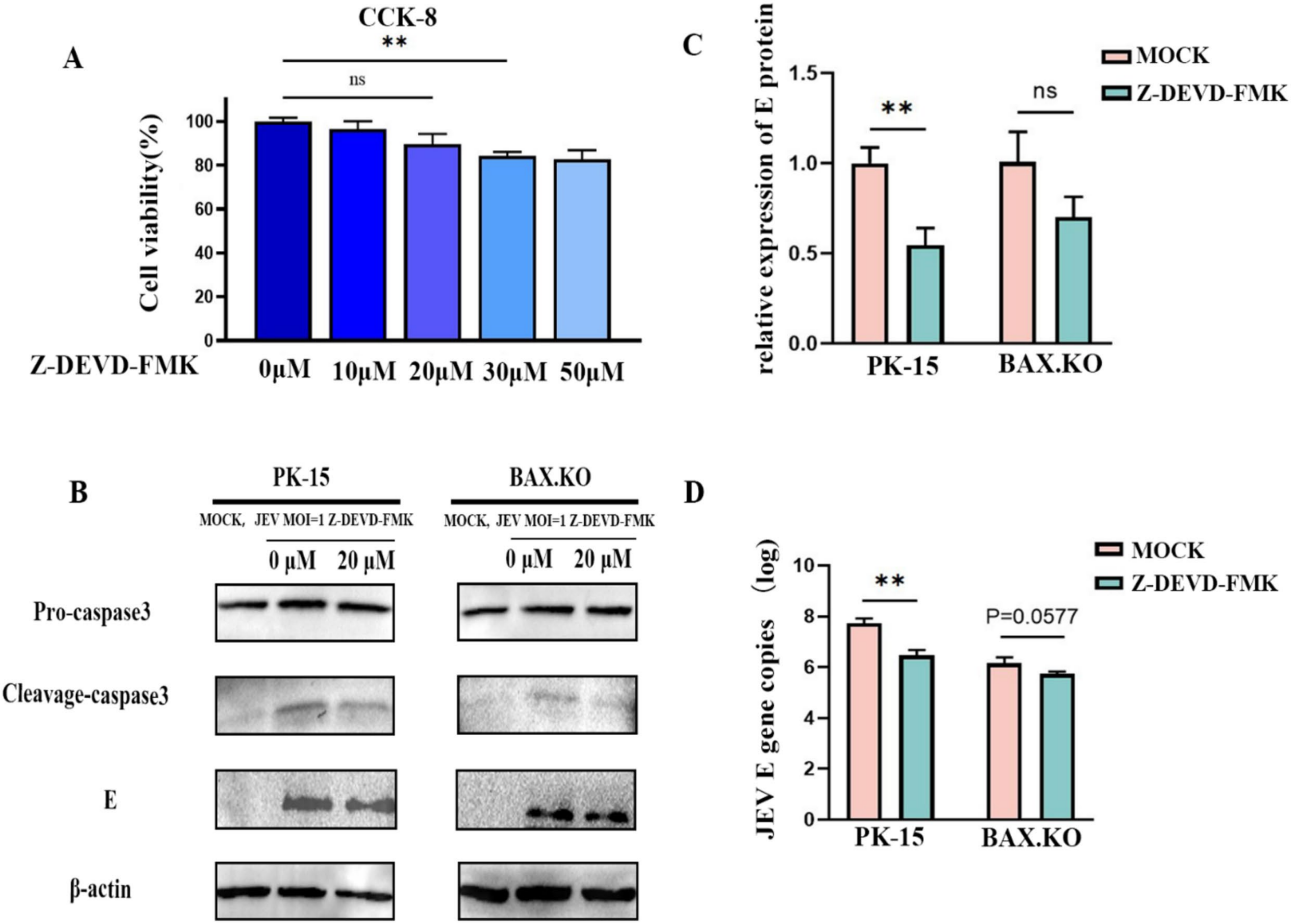
Japanese encephalitis virus (JEV) infection-induced apoptosis is important for viral proliferation and release. **(A)** PK-15 and BAX.KO cells were pre-incubated with different concentrations of Z-DEVD-FMK for 36 h, and then the appropriate amount of CCK8 solution was added. After incubation for 2 h, the appropriate inhibitor concentration was determined using a microplate reader. **(B)** PK-15 and BAX.KO cells were incubated with 20 μM Z-DEVD-FMK for 36 h, and the cells were infected with JEV for 36 h (multiplicity of infection [MOI] = 1). The viral load was analyzed using Western blotting. **(C)** E protein was quantified using ImageJ. **(D)** The amount of virus released into the culture medium was detected using real-time quantitative reverse transcription polymerase chain reaction (RT-qPCR). **p < 0.01. ns, not significant.

Caspase-3 inhibitor Z-DEVD-FMK with different concentrations was pre-incubated with PK-15 and BAX.KO cells for 36 h, and cell activity was detected by CCK8 to determine the optimal concentration of the inhibitor (Figure 7A). JEV-infected PK-15 and BAX.KO cells were pre-treated with Z-DEVD-FMK for 36 h (MOI = 1). E and caspase-3 proteins were detected using Western blotting (Figure 7B), and the JEV-E protein was quantitatively analyzed using ImageJ (Figure 7C). The culture fluid of the two cells was collected, and the virus content released into the culture fluid was detected using RT-qPCR. Inhibiting cell apoptosis can significantly inhibit the release of the virus outside the cell to achieve virus diffusion and proliferation (Figure 7D). In summary, inhibiting JEV-induced cell apoptosis can inhibit the proliferation of the virus in the cell and the release of the virus.

## 4 Discussion

Previous studies by our group have shown that the proapoptotic gene BAX may be involved in the JEV life cycle. Current research on BAX protein focuses on its ability to inhibit cancer cell proliferation by promoting apoptosis of cancer cells (Zhiqing et al., 2015). While some progress has been made on the correlation between other viruses and BAX protein, our search revealed that the relationship between JEV and BAX appears to be rarely reported. As the primary amplification host of JEV, we hypothesized that BAX also plays a pivotal role in the JEV invasion of porcine cells. To this end, we selected PK-15 cells, a commonly used research material for JEV, as the research object. PK-15 cells with BAX gene knockouts were constructed using CRISPR/Cas9. The overexpression of the BAX gene in PK-15 cells and the supplementation of the BAX gene in BAX.KO cells also demonstrated that BAX plays a positive regulatory role in the process of JEV proliferation (Figure 3). DAPI and Annexin V-FITC double staining experiments showed that BAX knockout significantly inhibited JEV-induced apoptosis (Figure 4).

Apoptosis is a crucial PCD pathway that is vital for maintaining tissue homeostasis, guiding embryonic development, and regulating immunity (Giuseppa et al., 2016; Ketelut-Carneiro and Fitzgerald, 2022). Viruses can regulate their own proliferation by promoting or inhibiting the apoptosis of host cells, which represents an essential aspect of the cell life cycle. For instance, inhibiting apoptosis caused by FeHV-1 infection with inhibitors can promote viral proliferation and increase viral titers, while inducing apoptosis leads to reduced viral protein expression, lower viral titers, and weakened autophagy, which may be related to the virus evading the host’s immune surveillance (Gianmarco et al., 2023a; Gianmarco et al., 2023b). IAV inhibits apoptosis by upregulating the anti-apoptotic phosphoinositide 3-kinase-protein kinase B (PI3 K-AKT) pathway in the early stage of infection while promoting apoptosis and viral proliferation by inhibiting this pathway and upregulating the proapoptotic p53 pathway in the late stage of infection (Gupta et al., 2017; Ampomah and Lim, 2020). Apoptosis is not the sole mechanism by which viruses induce cell death. Pyroptosis and necrosis constitute a synchronized, coordinated cell death system in which any one pathway can compensate for another, or all three pathways can operate in the same cell but at different moments depending on the context and timing (Pérez-Figueroa et al., 2021; Ketelut-Carneiro and Fitzgerald, 2022).

**Figure 8:**
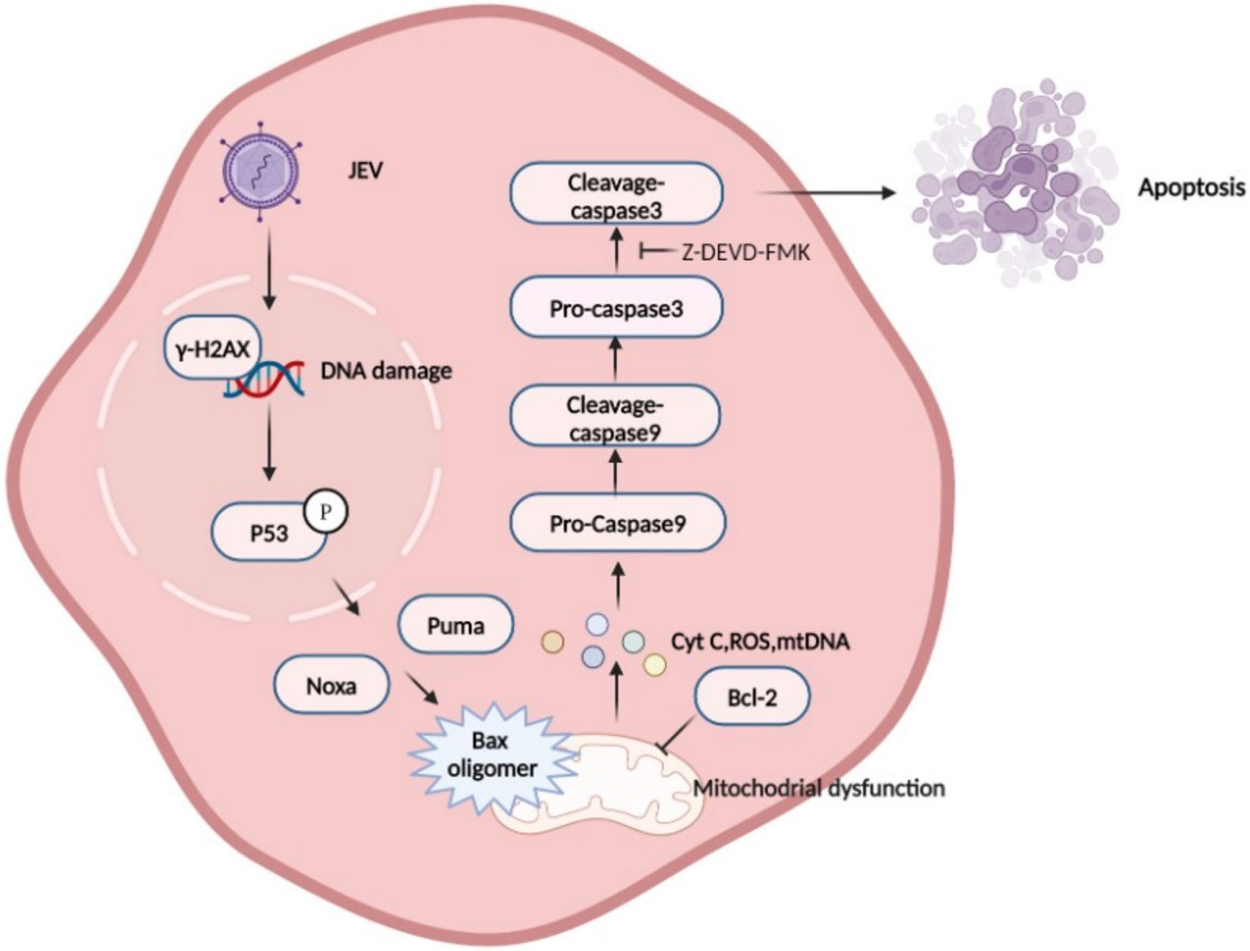
Schematic diagram of Japanese encephalitis virus (JEV)-mediated mitochondrial apoptosis pathway. JEV infection causes DNA damage, which promotes P53 phosphorylation in the nucleus. P53 induces a large number of genes involved in various steps of apoptosis signaling and execution (Todd et al., 2008), leading to mitochondrial membrane potential (MOMP), which is considered to be a key step in the mitochondrial apoptosis pathway. BAX is activated and inserted into the mitochondrial outer membrane and oligomerizes to form pores to allow Cytochrome C, mtDNA, and other proteins to be released from the membrane space. Mitochondria recruit and activate procaspase 9, which further activates executioner caspase 3 to induce apoptosis.

In the mitochondria-mediated apoptotic pathway, BAX is usually located in the cytoplasm or loosely bound to the mitochondrial outer membrane (MOM), whereas BAK is located predominantly in the outer mitochondrial membrane and remains inactive in non-apoptotic cells (Gitego et al., 2023). BAX-regulated MOMP is a key mechanism leading to cell death (Figure 8). Upon apoptotic stimulation, BAX induces MOMP by converting from inactive cytoplasmic monomers to lethal mitochondrial outer membrane oligomers. This process releases proapoptotic factors, such as cytochrome C and mtDNA, into the cytoplasm, facilitating apoptotic cysteine cascade signaling. Caspases play an important role in apoptosis, with caspase-9 being involved in the mitochondrial apoptotic pathway and activating the downstream protein caspase-3 for apoptosis. In this study, we found that JEV can activate both caspase-9 and caspase-3, and the proliferation and release of JEV was inhibited with a specific inhibitor of caspase-3 (Z-DEVD-FMK). This suggests that JEV can achieve viral proliferation and release through the mitochondrial apoptotic pathway. PK-15 cells lacking BAX expression showed resistance to JEV infection-induced apoptosis, providing evidence that JEV induces apoptosis by regulating BAX and revealing important clues to the mitochondrial permeability mechanism during JEV infection.

Mitochondria are present in almost all nucleated cells and are involved in apoptosis in addition to energy production. They contain their own DNA (mitochondrial DNA, or mtDNA), which encodes 13 essential mitochondrial proteins (Ayman et al., 2017). Studies have shown that some flavivirus infections release mtDNA (ZIKA, DENV) (Lai et al., 2018; Zheng et al., 2018). Apoptosis and mtDNA release require the activation of BAX and BAK, which release mtDNA through the megapores formed by BAX and BAK (Cosentino et al., 2022). The mtDNA binds to a wide variety of PAMPs, including Toll-like receptors (TLRs), inflammasomes, RIG-I-like receptors, and RIG-I-like receptors. Receptors (TLRs), inflammasomes, RNA sensors of the RIG-I-like receptor (RLR) family, and DNA sensors such as cGMP-AMP synthetase (cGAS), which in turn activate interferon α, interferon β, and/or nuclear factor-κB, as well as other signaling pathways that activate the type 1 interferon response and downstream ISGs to induce proinflammatory cytokines (Si Ming and Thirumala-Devi, 2015). These endogenous molecules are released under various stress conditions to activate the natural immune system. Our previous study showed that JEV infection in PK-15 cells activated the NLRP3 inflammatory pathway (Weimin et al., 2023). Here, we found that JEV infection leads to the release of mtDNA, which is subsequently activated by binding to PAMP. However, the precise mechanisms of action of mtDNA and PAMP remain to be investigated.

As a member of the Flaviviridae family and the Flavivirus genus (Hayes, 2009). JEV is closely related to West Nile virus (WNV), dengue virus (DENV), Zika virus (ZIKA), and other flaviviruses (Saiz et al., 2016). These flavivirus infections have been shown to activate multiple signaling pathways that can either activate or inhibit apoptosis in virus-infected cells, such as endoplasmic reticulum (ER) stress and the AKT/PI3K pathway (Toru et al., 2017; Wang et al., 2017; Yang et al., 2020). Current studies have shown that DENV, Zika, and WNV can all induce apoptosis through the mitochondrial apoptosis pathway (Hottz et al., 2013; Pan et al., 2021), which may be a common feature of members of the Flavivirus genus. However, there are still differences in the apoptosis induced by each flavivirus. Previous studies have shown that the ZIKV NS4B protein can regulate BAX recruitment and activation and trigger apoptotic programs (Xiaodong et al., 2021). During DENV infection, cell apoptosis can be regulated by depolarization of MOMP Δψ(m) through the expression and inhibition of BCL-2/BAX (Qi et al., 2015). The West Nile virus (WNV) capsid protein (WNVCp) binds HDM2 and sequesters it into the nucleolus, disrupting the formation of the HDM2 and p53 complex, leading to p53 stabilization and subsequent induction of mitochondrial dysfunction, and promoting apoptosis (Mi-Ran et al., 2007). In addition, many advances have been made in the research on anti-JEV infection. For example, the active ingredients of Arisaema plants show good binding affinity and stability to NS 5 and NS 3 helicases and can be used as candidates for anti-JEV drugs (Navyashree et al., 2021). Inhibition of mast cell-specific chymotrypsin can ultimately prevent JEV from entering the brain (Kant et al., 2021). How JEV participates in apoptosis and whether it interacts with apoptotic proteins remains unknown, necessitating further studies to explore these possibilities.

## 5 Conclusion

In conclusion, the results of this study indicate that JEV induces apoptosis of PK-15 cells through the mitochondrial apoptosis pathway. BAX is an important host protein in the process of JEV-induced mitochondrial apoptosis. JEV activates downstream caspase enzymes through the P53-BAX axis, promoting mitochondrial apoptosis and facilitating viral proliferation and release. However, it is not yet known whether there are other mechanisms of JEV-induced cell death beyond the mitochondrial apoptosis pathway. Further research is required to explore this possibility. The current state of research on JEV-induced death modes is incomplete, but the exploration of JEV and BAX in this study represents significant progress in this field.

## Data Availability

The raw data supporting the conclusions of this article will be made available by the authors, without undue reservation.

## Footnotes

^1^http://chopchop.cbu.uib.no/

## References

[ref1] Ampomah P. Lim L. (2020). Influenza a virus-induced apoptosis and virus propagation. Apoptosis 25, 1–11. doi: 10.1007/s10495-019-01575-3, PMID: 31667646

[ref2] Avi A. Guy S. (2014). Regulated cell death: signaling and mechanisms. Annu. Rev. Cell Dev. Biol. 30, 337–356. doi: 10.1146/annurev-cellbio-100913-013226 25150011

[ref3] Ayman W. E.-H. William J. C. Fernando S. (2017). Mitochondrial DNA maintenance defects. Biochim. Biophys. Acta Mol. Basis Dis. 1863, 1539–1555. doi: 10.1016/j.bbadis.2017.02.017 28215579

[ref4] Bimmi S. David G. Michael S. D. (2003). Infection and injury of neurons by West Nile encephalitis virus. J. Virol. 77, 13203–13213. doi: 10.1128/JVI.77.24.13203-13213.2003 14645577 PMC296085

[ref5] Carneiro B. El-Deiry W. (2020). Targeting apoptosis in cancer therapy. Nat. Rev. Clin. Oncol. 17, 395–417. doi: 10.1038/s41571-020-0341-y, PMID: 32203277 PMC8211386

[ref6] Caroppi P. Sinibaldi F. Fiorucci L. Santucci R. (2009). Apoptosis and human diseases: mitochondrion damage and lethal role of released cytochrome C as proapoptotic protein. Curr. Med. Chem. 16, 4058–4065. doi: 10.2174/092986709789378206, PMID: 19754424

[ref7] Cosentino K. Hertlein V. Jenner A. Dellmann T. Gojkovic M. Peña-Blanco A.. (2022). The interplay between BAX and BAK tunes apoptotic pore growth to control mitochondrial-DNA-mediated inflammation. Mol. Cell 82, 933–949.e9. doi: 10.1016/j.molcel.2022.01.008, PMID: 35120587 PMC8901441

[ref8] Czabotar P. Westphal D. Dewson G. Ma S. Hockings C. Fairlie W.. (2013). Bax crystal structures reveal how BH3 domains activate Bax and nucleate its oligomerization to induce apoptosis. Cell 152, 519–531. doi: 10.1016/j.cell.2012.12.031, PMID: 23374347

[ref9] Damien B. Eicke L. Bernardo S. F. (2021). Necroptosis, pyroptosis and apoptosis: an intricate game of cell death. Cell. Mol. Immunol. 18, 1106–1121. doi: 10.1038/s41423-020-00630-3 33785842 PMC8008022

[ref10] Gianmarco F. Consiglia L. Maria Francesca S. Brunella R. Giuseppe I. Roberto C.. (2023a). Apoptosis is mediated by FeHV-1 through the intrinsic pathway and interacts with the autophagic process. Virol. J. 20:295. doi: 10.1186/s12985-023-02267-w 38087282 PMC10716993

[ref11] Gianmarco F. Consiglia L. Sara D. Roberto C. Ugo P. Serena M. (2023b). Modifications of the PI3K/Akt/mTOR axis during FeHV-1 infection in permissive cells. Front. Vet. Sci. 10:1157350. doi: 10.3389/fvets.2023.1157350 37026095 PMC10072329

[ref12] Gitego N. Agianian B. Mak O. Kumar Mv V. Cheng E. Gavathiotis E. (2023). Chemical modulation of cytosolic BAX homodimer potentiates BAX activation and apoptosis. Nat. Commun. 14:8381. doi: 10.1038/s41467-023-44084-3, PMID: 38104127 PMC10725471

[ref13] Giuseppa P. Daniela T. Claudia C. Alessia G. Gabriella D. O. (2016). Apoptosis as anticancer mechanism: function and dysfunction of its modulators and targeted therapeutic strategies. Aging (Albany NY) 8, 603–619. doi: 10.18632/aging.100934 27019364 PMC4925817

[ref14] Gupta A. Bharadwaj M. Kumar A. Mehrotra R. (2017). Spiro-oxindoles as a promising class of small molecule inhibitors of p53-MDM2 interaction useful in targeted Cancer therapy. Top. Curr. Chem. 375:3. doi: 10.1007/s41061-016-0089-0, PMID: 27943171

[ref15] Hayes E. (2009). Zika virus outside Africa. Emerg. Infect. Dis. 15, 1347–1350. doi: 10.3201/eid1509.090442, PMID: 19788800 PMC2819875

[ref16] Hottz E. Oliveira M. Nunes P. Nogueira R. Valls-De-Souza R. Da Poian A.. (2013). Dengue induces platelet activation, mitochondrial dysfunction and cell death through mechanisms that involve DC-SIGN and caspases. J. Thromb. Haemost. 11, 951–962. doi: 10.1111/jth.12178, PMID: 23433144 PMC3971842

[ref17] James C. P. Tristan P. L. Benjamin J. L. James G. L. (2018). Japanese encephalitis: the vectors, ecology and potential for expansion. J. Travel Med. 25, S16–S26. doi: 10.1093/jtm/tay009 29718435

[ref18] Junqi H. Ying L. Yiming Q. Yingke Z. Lin Z. Zilian W.. (2014). Coordinated regulation of autophagy and apoptosis determines endothelial cell fate during Dengue virus type 2 infection. Mol. Cell. Biochem. 397, 157–165. doi: 10.1007/s11010-014-2183-3 25138703

[ref19] Kant K. Rawat R. Bhati V. Bhosale S. Sharma D. Banerjee S.. (2021). Computational identification of natural product leads that inhibit mast cell chymase: an exclusive plausible treatment for Japanese encephalitis. J. Biomol. Struct. Dyn. 39, 1203–1212. doi: 10.1080/07391102.2020.1726820, PMID: 32036760

[ref20] Ketelut-Carneiro N. Fitzgerald K. (2022). Apoptosis, pyroptosis, and necroptosis-oh my! The many ways a cell can die. J. Mol. Biol. 434:167378. doi: 10.1016/j.jmb.2021.167378, PMID: 34838807

[ref21] Kurt D. Ramya S. Tullia L. Craig T. Eileen W. (2002). Bax and Bak independently promote cytochrome C release from mitochondria. J. Biol. Chem. 277, 14127–14134. doi: 10.1074/jbc.M109939200 11836241

[ref22] Lai J. Wang M. Huang C. Wu C. Hung L. Yang C.. (2018). Infection with the dengue RNA virus activates TLR9 signaling in human dendritic cells. EMBO Rep. 19:e46182. doi: 10.15252/embr.201846182, PMID: 29880709 PMC6073071

[ref23] Liying Z. Donald C. C. (2008). Dynamics and structure of the Bax-Bak complex responsible for releasing mitochondrial proteins during apoptosis. J. Cell Sci. 121, 2186–2196. doi: 10.1242/jcs.024703 18544634

[ref24] Lopez A. Aldaba J. Roque V. Tandoc A. Sy A. Espino F.. (2015). Epidemiology of Japanese encephalitis in the Philippines: a systematic review. PLoS Negl. Trop. Dis. 9:e0003630. doi: 10.1371/journal.pntd.0003630, PMID: 25794009 PMC4367992

[ref25] Lorenzo G. Catherine B. Eugenia M. Zahia T. Guido K. (2008). Viral control of mitochondrial apoptosis. PLoS Pathog. 4:e1000018. doi: 10.1371/journal.ppat.1000018 18516228 PMC2376094

[ref26] Lukasz P. S. Dong-Hoon C. Austin P. Taylor H. Nolan L. B. Michal H. (2017). Ribosomal stress and Tp53-mediated neuronal apoptosis in response to capsid protein of the Zika virus. Sci. Rep. 7:16652. doi: 10.1038/s41598-017-16952-8 29192272 PMC5709411

[ref27] Mark P. A. L.-V. Jerry E. C. (2015). The deadly landscape of pro-apoptotic BCL-2 proteins in the outer mitochondrial membrane. FEBS J. 283, 2676–2689. doi: 10.1111/febs.13624 PMC4907887 26662859

[ref28] Mi-Ran Y. Sung Ryul L. Wonkyung O. Eun-Woo L. Jung-Yong Y. Jin-Ju N.. (2007). West Nile virus capsid protein induces p53-mediated apoptosis via the sequestration of HDM2 to the nucleolus. Cell. Microbiol. 10, 165–176. doi: 10.1111/j.1462-5822.2007.01027.x 17697133 PMC7162166

[ref29] Misra U. Kalita J. (2010). Overview: Japanese encephalitis. Prog. Neurobiol. 91, 108–120. doi: 10.1016/j.pneurobio.2010.01.008 20132860

[ref30] Navyashree V. Kant K. Kumar A. (2021). ArisaemaNatural chemical entities from genus might be a promising break-through against Japanese encephalitis virus infection: a molecular docking and dynamics approach. J. Biomol. Struct. Dyn. 39, 1404–1416. doi: 10.1080/07391102.2020.1731603, PMID: 32072856

[ref31] Pan Y. Cheng A. Wang M. Yin Z. Jia R. (2021). The dual regulation of apoptosis by Flavivirus. Front. Microbiol. 12:654494. doi: 10.3389/fmicb.2021.654494, PMID: 33841381 PMC8024479

[ref32] Pérez-Figueroa E. Lvarez-Carrasco P. Ortega E. Maldonado-Bernal C. (2021). Neutrophils: many ways to die. Front. Immunol. 12:631821. doi: 10.3389/fimmu.2021.631821, PMID: 33746968 PMC7969520

[ref33] Peter G. E. K. (2015). Viruses, apoptosis, and neuroinflammation—a double-edged sword. J. Neurovirol. 21, 1–7. doi: 10.1007/s13365-014-0306-y 25604493

[ref34] Qi Y. Li Y. Zhang Y. Zhang L. Wang Z. Zhang X.. (2015). IFI6 inhibits apoptosis via mitochondrial-dependent pathway in dengue virus 2 infected vascular endothelial cells. PLoS One 10:e0132743. doi: 10.1371/journal.pone.0132743, PMID: 26244642 PMC4526556

[ref35] Quan Z. Jiaqi L. Zhaoyang L. Yucan Z. Shaopo Z. Xueyan D.. (2023). Japanese encephalitis virus NS4B inhibits interferon beta production by targeting TLR3 and TRIF. Vet. Microbiol. 284:109849. doi: 10.1016/j.vetmic.2023.109849 37597377

[ref36] Saiz J. Vázquez-Calvo Á. Blázquez A. B. Merino-Ramos T. Escribano-Romero E. Martín-Acebes M. (2016). Zika virus: the latest newcomer. Front. Microbiol. 7:496. doi: 10.3389/fmicb.2016.00496 27148186 PMC4835484

[ref37] Shanna Q. Zhong W. Wanting Y. Jinling H. Yinfeng Y. Jinghui W. (2022). The role of BCL-2 family proteins in regulating apoptosis and cancer therapy. Front. Oncol. 12:985363. doi: 10.3389/fonc.2022.985363 36313628 PMC9597512

[ref38] Sharma K. Vrati S. Kalia M. (2021). Pathobiology of Japanese encephalitis virus infection. Mol. Asp. Med. 81:100994. doi: 10.1016/j.mam.2021.100994, PMID: 34274157

[ref39] Si Ming M. Thirumala-Devi K. (2015). Converging roles of caspases in inflammasome activation, cell death and innate immunity. Nat. Rev. Immunol. 16, 7–21. doi: 10.1038/nri.2015.7 26655628 PMC4915362

[ref40] So Lee P. Yan-Jang S. H. Amy C. L. Victoria B. A. Susan M. H. Scott M D.. (2018). North American domestic pigs are susceptible to experimental infection with Japanese encephalitis virus. Sci. Rep. 8:7951. doi: 10.1038/s41598-018-26208-8 29784969 PMC5962597

[ref41] Solomon T. (2004). Flavivirus encephalitis. N. Engl. J. Med. 351, 370–378. doi: 10.1056/NEJMra030476 15269317

[ref42] Solomon T. Dung N. Kneen R. Gainsborough M. Vaughn D. Khanh V. (2000). Japanese encephalitis. J. Neurol. Neurosurg. Psychiatry 68, 405–415. doi: 10.1136/jnnp.68.4.405, PMID: 10727474 PMC1736874

[ref43] Suzuki T. Okamoto T. Katoh H. Sugiyama Y. Kusakabe S. Tokunaga M.. (2018). Infection with flaviviruses requires BCLXL for cell survival. PLoS Pathog. 14:e1007299. doi: 10.1371/journal.ppat.1007299, PMID: 30261081 PMC6177207

[ref44] Todd R. Eduardo S. Patricia C. Arnold L. (2008). Transcriptional control of human p53-regulated genes. Nat. Rev. Mol. Cell Biol. 9, 402–412. doi: 10.1038/nrm2395 18431400

[ref45] Toru O. Tatsuya S. Shinji K. Makoto T. Junki H. Yuka M.. (2017). Regulation of apoptosis during flavivirus infection. Viruses 9:243. doi: 10.3390/v9090243 28846635 PMC5618009

[ref46] Wang L. Yang L. Fikrig E. Wang P. (2017). An essential role of PI3K in the control of West Nile virus infection. Sci. Rep. 7:3724. doi: 10.1038/s41598-017-03912-5, PMID: 28623344 PMC5473900

[ref47] Weimin X. Ke Y. Yi Z. Sanjie C. Qigui Y. Xiaobo H.. (2023). BAK-mediated pyroptosis promotes Japanese encephalitis virus proliferation in porcine kidney 15 cells. Viruses 15:974. doi: 10.3390/v15040974 37112954 PMC10142372

[ref48] Wood D. E. Newcomb E. W. (2000). Cleavage of Bax enhances its cell death function. Exp. Cell Res. 256, 578–582. doi: 10.1006/excr.2000.4859, PMID: 10772810

[ref49] Xiaodong H. Jiuqiang W. Yang Y. Shuxiang Q. Fang W. Ziyi Z.. (2021). Zika virus infection induced apoptosis by modulating the recruitment and activation of pro-apoptotic protein Bax. J. Virol. 95:e01445-20. doi: 10.1128/JVI.01445-20 PMC8103684 33536166

[ref50] Xuefang C. Xingming D. Stratford May W. (2003). Cleavage of Bax to p18 Bax accelerates stress-induced apoptosis, and a cathepsin-like protease may rapidly degrade p18 Bax. Blood 102, 2605–2614. doi: 10.1182/blood-2003-01-0211 12816867

[ref51] Yang S. Gorshkov K. Lee E. Xu M. Cheng Y. Sun N.. (2020). Zika virus-induced neuronal apoptosis via increased mitochondrial fragmentation. Front. Microbiol. 11:598203. doi: 10.3389/fmicb.2020.598203, PMID: 33424801 PMC7785723

[ref52] Zheng Y. Liu Q. Wu Y. Ma L. Zhang Z. Liu T.. (2018). Zika virus elicits inflammation to evade antiviral response by cleaving cGAS via NS1-caspase-1 axis. EMBO J. 37:e99347. doi: 10.15252/embj.201899347, PMID: 30065070 PMC6138430

[ref53] Zhiqing L. Ye D. Na Y. Christopher W. Haiying C. Jia Z. (2015). Direct activation of Bax protein for cancer therapy. Med. Res. Rev. 36, 313–341. doi: 10.1002/med.21379 26395559 PMC4752390

